# Colonic electrical stimulation promotes colonic motility through regeneration of myenteric plexus neurons in slow transit constipation beagles

**DOI:** 10.1042/BSR20182405

**Published:** 2019-05-17

**Authors:** Yongbin Wang, Qian Wang, Kudelaidi Kuerban, Mengxue Dong, Feilong Qi, Gang Li, Jie Ling, Wei Qiu, Wenzhong Zhang, Li Ye

**Affiliations:** 1 Pudong New Area People’s Hospital Affiliated to Shanghai University of Medicine and Health Sciences, Shanghai 201200, China; 2 Department of Microbiological and Biochemical Pharmacy, School of Pharmacy, Fudan University, Shanghai 201203, China; 3 Department of Pathology, Shanghai Cancer Center, Fudan University, Shanghai 200032, China

**Keywords:** colonic electrical stimulation, enteric nervous system, myenteric plexus, slow transit constipation, synaptophysin

## Abstract

Slow transit constipation (STC) is a common disease characterized by markedly delayed colonic transit time as a result of colonic motility dysfunction. It is well established that STC is mostly caused by disorders of relevant nerves, especially the enteric nervous system (ENS). Colonic electrical stimulation (CES) has been regarded as a valuable alternative for the treatment of STC. However, little report focuses on the underlying nervous mechanism to normalize the delayed colonic emptying and relieve symptoms. In the present study, the therapeutic effect and the influence on ENS triggered by CES were investigated in STC beagles. The STC beagle model was established by oral administration of diphenoxylate/atropine and alosetron. Histopathology, electron microscopy, immunohistochemistry, Western blot analysis and immunofluorescence were used to evaluate the influence of pulse train CES on myenteric plexus neurons. After 5 weeks of treatment, CES could enhance the colonic electromyogram (EMG) signal to promote colonic motility, thereby improving the colonic content emptying of STC beagles. HE staining and transmission electron microscopy confirmed that CES could regenerate ganglia and synaptic vesicles in the myenteric plexus. Immunohistochemical staining showed that synaptophysin (SYP), protein gene product 9.5 (PGP9.5), cathepsin D (CAD) and S-100B in the colonic intramuscular layer were up-regulated by CES. Western blot analysis and immunofluorescence further proved that CES induced the protein expression of SYP and PGP9.5. Taken together, pulse train CES could induce the regeneration of myenteric plexus neurons, thereby promoting the colonic motility in STC beagles.

## Introduction

Chronic constipation, a functional bowel disorder, affects approximately 14% of adults worldwide [1]. Slow transit constipation (STC) is the major cause of chronic constipation which is characterized by markedly prolonged colonic transit time as a result of the colonic motility function disorder [2,3]. Usually, patients with STC suffer from a common sense of abdominal pain, nausea, depression and sickness, which seriously influence their social ability and health-related quality of life [4–6]. Current clinical treatments include cathartics, prokinetics and aggressive surgery which can increase bowel movement frequency to a certain degree. However, pharmacological interventions is prone to drug dependency and relapse after drug withdrawal [3]. Surgical treatments such as subtotal colectomy and total colectomy in STC patients may adversely affect the quality of life due to the risk of postoperative diarrhea or incontinence, and result in a heavy healthcare burden [7–9].

The enteric nervous system (ENS), located in the intestinal wall, regulates various functions including contraction of intestine, homeostasis and blood flow [10]. As the ‘second brain’, the ENS contains large amounts of neurons working independently from the central nervous system [11]. Researches have identified that STCs are mostly caused by disorders of the relevant nerves, especially the ENS [12,13]. Direct electrical stimulation has been explored in stomach [14], small intestine [15] and colon [16] to overcome the abnormal gastroenteric motility. These experiments showed promising results in the vertebrates. However, such investigation was mostly carried out in mice and chicken. Colonic electrical stimulation (CES), a valuable alternative for the treatment of STC, was reported to improve the colon motility by adjusting the bioelectrical activity in animal models or patients with STC [17]. However, little report focuses on the underlying nervous mechanism to normalize the delayed colonic emptying and relieve symptoms. We hypothesized that CES may also repair the disorders of the relevant nerves and then improve the colonic motility.

The frequently selected colonic stimulation sites localized at the proximal colon [18–20], the descending colon [21,22] and the rectosigmoid junction (RSJ) [23,24]. Extensive researches [25] have concluded that long-pulse stimulation is inferior to pulse train stimulation in emptying the intestinal transit content. Therefore, we employed pulse train stimulation and implanted electrodes at the proximal colon in dogs.

The aim of the present study was to uncover the function of CES in canine models of delayed colonic transit and the changes in the myenteric plexus neurons. First, the STC beagle models were established. After CES treatment, we observed the colonic transit time of the sham treatment group was longer than that of CES treatment and control groups, and electrical stimulation significantly enhanced the colonic electromyogram (EMG) signal. More importantly, histopathology and TEM analysis showed increased ganglia and synaptic vesicles existing in the colon myenteric plexus of the CES treatment group as compared with that of the sham CES group. We also employed immunohistochemistry, immunofluorescence and Western blot to detect the changes of intestinal nerve related proteins such as synaptophysin (SYP), protein gene product 9.5 (PGP9.5), cathepsin D (CAD) and S-100B. Our results suggested that CES might reduce the degeneration of the myenteric plexus neurons, thereby contributing to the therapeutic effect on STC beagles.

## Materials and methods

### Materials

Nine healthy, female beagles (weighing 16.2 ± 2.6 kg) were purchased from School of Agriculture and Biology at Shanghai Jiao Tong University, and housed in isolator cages. The electrical stimulators consisting of programming stick, pulser and a pair of wired electrodes were obtained from Rishenan Technology Development Co. Ltd., Changzhou, Jiangsu, China. Propofol Injection was obtained from Xi’an Libang Pharmaceutical Co. Ltd. Seramine Hydrochloride Injection was purchased from Jilin Animal Husbandry Animal Health Products Co. Ltd. Diphenoxylate was purchased from Changzhou Kangpu Pharmaceutical Co. Ltd. Alosetron hydrochloride was purchased from Hubei Jusheng Technology Co. Ltd. The antibodies including S100B and CAD were purchased from Thermo Fisher Scientific (Rockford, U.S.A.). PGP9.5 antibody was purchased from Abcam (ab8189). SYP antibody was purchased from Genway (GWB-MQ104E).

### Surgical procedure and EMG signal detection

All experiments were approved by the Animal Care and Use Committee of School of Pharmacy at Fudan University. Six healthy female beagles were injected intravenously with the combination of Propofol injection (1.5 ml/kg) and Seramine Hydrochloride injection (2.5 ml/kg). Then a midline laparotomy under general anesthesia was performed. A pair of wired bipolar electrodes was implanted in the seromuscular layer at 1.0 cm distal to the ileocecal junction. Next, we implanted the pulser in subcutaneous pocket created along the right side of the midabdominal incision, and then connected it to the electrodes.

**Figure 1:**
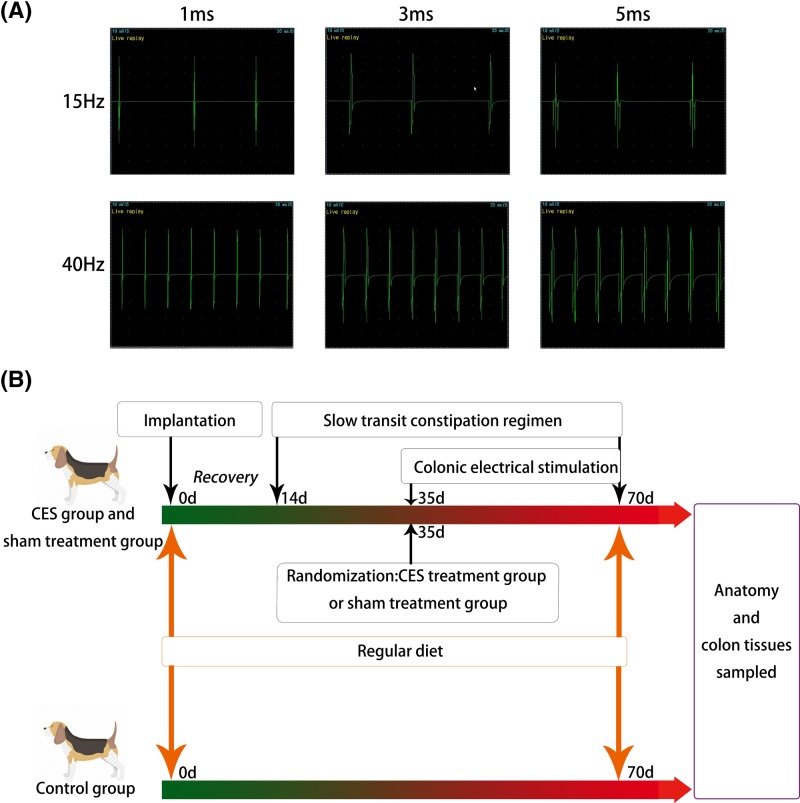
Experiment procedure and EMG signal detection The pulse signals detected by Full-function Electromyography (**A**) and the experiment flowchart (**B**). (A) After the electrodes were implanted in the seromuscular layer and connected to the pulser, the pulse signals were detected as described in ‘Materials and methods’ when the pulse width was set at 1, 3 and 5 ms, and frequency at 15 and 40 Hz by programming stick respectively. (B) After recovering from implantation, dogs were started on the STC regimen. Three weeks later, the STC beagles were randomized to receive either stimulation or sham stimulation, with another three dogs without implanted electrodes as control. During the experiment, all groups received regular diet unless otherwise specified. At the end of the experiment, all dogs were anatomized and the colon tissues were sampled for various analyses.

A Full-function Electromyography (Keypoint Portable 33A7, Alpine BioMed ApS) was employed to detect the pulse signals generated by the electrical stimulator which was controlled by programming stick *in vitro* after the electrodes and pulser were implanted. In detail, a pair of detection electrodes was stitched in the colonic wall at 5.0 cm distal to the stimulation electrodes with an interval of 0.5–1.0 cm between the positive and the negative electrodes. When the pulse width was set at 1, 3 and 5 ms, and frequency at 15 and 40 Hz respectively, the pulse signals were detected by the Full-function Electromyography. The results in Figure 1A proved that the electrical stimulator was in working order. After surgery, the dogs were fasted for 1 day, and then sufficient standard solid food and water were supplied at 8:00 a.m. each day. Antibiotics were taken orally to protect them from infection. Two weeks later, all beagles recovered from surgery completely.

### STC beagle model and CES treatment

The experiment flowchart was shown in Figure 1B. Following 2 weeks recovery period after the operation, six beagles were fed with 3 mg/kg/day compound diphenoxylate (1 mg compound diphenoxylate contains 0.01 mg atropine sulfate) and 5 mg/day alosetron hydrochloride [16]. After 5 days of medication, the animals began to show infrequent bowel movements and dry stools. Three weeks later, 20 radiopaque markers (in a capsule) were given through mouth and the establishment of STC model was confirmed by abdominal X-rays. Then STC beagles were randomly divided into two groups, CES treatment group and sham treatment group, with another three beagles without implanted electrodes as control group. Beagles in the CES treatment group were stimulated by programming stick at 8:00 a.m. everyday, and the gastrointestinal transit and defecation were simultaneously evaluated in all groups during this period. After 5 weeks of stimulation, the colonic EMG signal was detected as described above, and all animals were anatomized and the colon tissues were removed for investigation of myenteric plexus neurons.

### Histopathology

After beagles were killed, colon tissues were immediately fixed in 4% paraformaldehyde solution for 24 h, then paraffin-embedded and sectioned at 4 µm. At least ten randomly selected colon histologic sections in each group were stained with Hematoxylin–Eosin (H&E) for general morphological analysis, and examined by light microscopy to detect the alteration of histological structures.

### Electron microscopy

The colon tissues were fixed for 4 h in 2.5% glutaraldehyde fixative (pH 7.4). After four rinses in 0.05 mol/l cacodylate buffer containing 0.22 mol/l sucrose, full-thickness strips (10 × 3 mm) were fixed in 1% phosphate-buffered OsO_4_ (pH 7.4) and embedded in Epon after dehydration with acetone. Ultramicrotome was used to obtain semi-thin sections which were then stained with Toluidine Blue solution in 0.1 mol/l borate buffer, thereafter observed by a light microscope at select target areas. The selected areas were sliced into ultra-thin sections using a diamond knife with ultramicrotome, and then stained with a saturated uranyl acetate solution in methanol (50:50) at 45°C for 12 min, followed by a concentrated bismuth subnitrate aqueous solution at room temperature for 10 min. These sections were observed at 80 kV through a JEM 1410 transmission electron microscope and photographed (JEOL, Inc., U.S.A.).

### Immunohistochemistry

The colon tissues were fixed in 4% paraformaldehyde solution, paraffin-embedded and sliced into sections as described above. The sections were then processed for immunohistochemistry after de-paraffinizing. Briefly, after 30-min incubation in 3% hydrogen peroxide to block endogenous peroxidase, the sections were incubated with primary antibody anti-SYP, anti-PGP9.5, anti-S100B or anti-CAD at 4°C for overnight. The slides were washed in PBS and incubated for 1.5 h with HRP-conjugated secondary antibody (goat anti-rabbit IgG, DAKO) at room temperature. After washing with PBS, the sections were incubated for 10 min with 3,3′-diaminobenzidine (DAB) chromogenic solution followed by washing in PBS. The slides were counterstained with Hematoxylin and dehydrated. The immunopositive areas displayed brown, and the nuclei were stained blue by Hematoxylin. The antibody specificity was confirmed by negative control without primary antibody treatment. Immunopositive areas were detected by an inverted microscope (Nikon, Japan). ImageJ software was used to perform result quantitation.

### Western blot analysis

The colon tissues were homogenized after addition of RIPA Lysis Buffer and PMSF, and then centrifuged (12000×***g***, 4°C) for 10 min to obtain the supernatant. After the protein concentration was determined by the bicinchoninic acid (BCA) method, equal amount of total protein per lane was electrophoretically separated by sodium dodecyl sulfate/polyacrylamide gel electrophoresis (SDS/PAGE), and then transferred to polyvinylidene fluoride (PVDF) membranes. The membranes were blocked with 3% bovine serum albumin (BSA) powder in 0.05% Tris-buffered saline and Tween 20 (TBST) for 1 h at room temperature and then incubated overnight at 4°C with anti-SYP primary antibody. After overnight incubation, the membranes were washed for three times and then incubated for 2 h at room temperature with peroxidase–conjugated secondary antibodies. Detection was performed with enhanced chemiluminescence reagents (Pierce, Rockford, IL, U.S.A.). Intensities in the resulting bands were quantitated by IQuantTL software (GE Healthcare, U.S.A.).

### Immunofluorescence

After blocking for 1 h at room temperature with 0.1% sodium azide-PBS containing Triton-X 100 (0.5%) and BSA (4%), the tissues were incubated overnight at room temperature with the PGP9.5 antibodies diluted in the blocking solution, washed three times, and further incubated for 1.5 h at room temperature with the secondary antibodies. Finally, the tissues were washed twice, incubated with nuclear dye DAPI solution for 5 min at room temperature, and washed twice. The tissues were immediately analyzed by confocal microscopy (Nikon, Japan). The immunopositive areas displayed green fluorescence, and the nuclei showed DAPI’s blue fluorescence. The quantitative analysis of the immunopositive areas was performed by ImageJ software.

### Statistical analysis

GraphPad Prism 5 was employed for statistical analysis and data from the present study were presented as mean values with standard deviations (S.D.). The statistical significance of the differences between groups was evaluated by Student’s *t* test. *, **, ***, and **** indicated *P*<0.05, *P*<0.01, *P*<0.001 and *P*<0.0001, respectively.

## Results

### CES enhanced colonic content emptying of STC beagles

**Figure 2:**
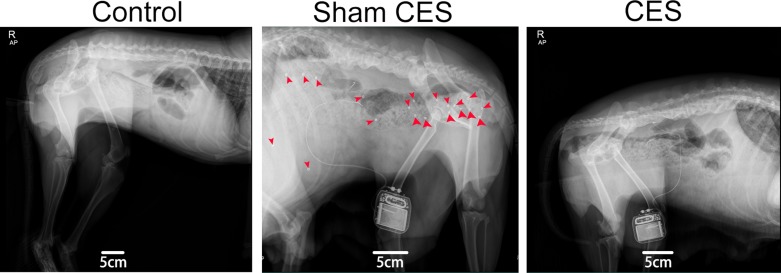
Representative abdominal X-ray images of beagles at 30 h after oral administration of radiopaque markers The sites of the radiopaque markers were indicated by the red arrows. After 5 weeks of CES treatment, animals in the CES and control groups had no residual radiopaque markers, while those in the sham CES group kept almost all radiopaque markers in abdomen.

Three weeks after the first feeding of diphenoxylate and alosetron, beagles’ defecating frequency changed from two times everyday to once every 2 days, and feces were obviously dryer. At 30 h after the oral administration of radiopaque markers, abdominal X-rays displayed that most of radiopaque markers remained in the abdomen, which confirmed the establishment of STC beagle model. Beagles in the CES treatment group were stimulated for 30 min by programming stick once a day at 8:00 a.m., and the parameters of pulse train stimulation were set as: 2s-pulse on, 3s-pulse off, 40 Hz and pulse width 3 ms according to beagles’ behavior (the three beagles in the CES treatment group showed no obvious sign of pain or discomfort during the electrical stimulation). As shown in Figure 2, after 5 weeks of CES treatment, the beagles in the control and CES groups had no residual radiopaque markers, while those in the sham CES group kept almost all radiopaque markers in abdomen, which meant the colonic transit time of control and CES groups was significantly shorter than that of sham CES group. Meanwhile, the defecating frequency and the feces characteristics of STC beagles returned to normal after CES treatment. The result indicated that CES could improve the symptoms of STC.

### The colonic EMG signal was strongly promoted by CES

**Figure 3:**
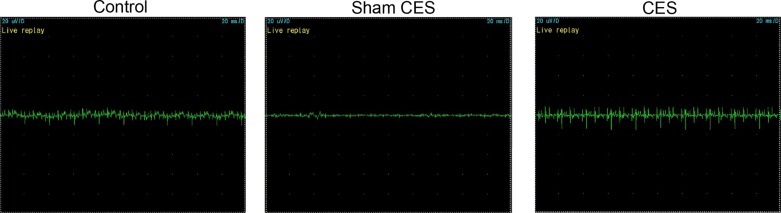
The colonic EMG signal of beagles detected by Full-function EMG After 5 weeks of CES treatment, the colonic EMG signal of animals with STC was remarkably enhanced by CES. The scales of the signal magnitude in all three figures were 20 μV/D and the scales of the time were 20 ms/D.

To further evaluate the therapeutic efficiency of CES on STC beagles, the beagle’s colon EMG signal was measured after 5 weeks of treatment. Results displayed that CES could indeed influence the colonic EMG signal. Especially, the colonic EMG signal of the beagles with STC was remarkably enhanced by CES (Figure 3), indicating that CES could not only improve the colonic content emptying, but also enhance the EMG signal to promote colonic motility.

### Effects of CES on the number of ganglia in colon tissue

**Figure 4:**
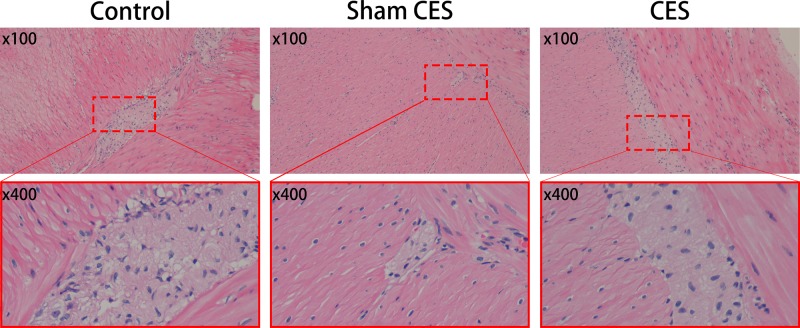
Colon myenteric plexus detected by HE staining The sites and area of the myenteric plexus tracts were indicated by the red pane. After 5 weeks of the CES treatment, there were a large number of ganglia between the circular and longitudinal muscles in the colon tissues of both CES group and control group, while significantly degenerated myenteric plexus was observed in the sham CES group. A large number of neurons with big round nuclei and nucleolus were also observed in the myenteric plexus in both control and CES groups.

The myenteric plexus, existing between the circular and longitudinal muscles, is crucial for the normal functioning of the intestinal tract. Degeneration of myenteric plexus can lead to intestinal motor dysfunction, causing secondary constipation and other diseases. As shown in Figure 4, HE staining was performed to illustrate the characteristics of colon myenteric plexus in the three groups of beagles. There were a large number of ganglia between the circular and longitudinal muscles in the colon tissues of both CES group and control group, while significantly degenerated myenteric plexus was observed in the sham CES group. Moreover, a great deal of large neuron cell bodies with big round nuclei and clear nucleolus were observed in the myenteric plexus in both control and CES groups.

### Effects of CES on ultrastructural features of colonic myenteric plexus neurons

**Figure 5:**
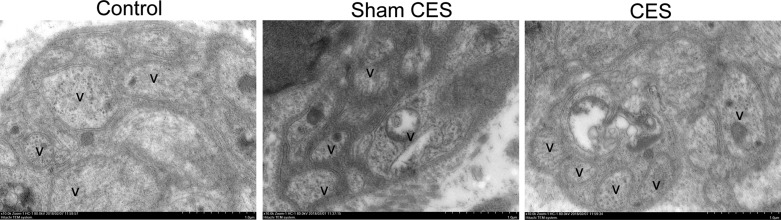
Representative ultrastructure of the colon myenteric plexus neurons observed by transmission electron microscopy After 5 weeks of treatment, CES increased the synaptic vesicles in the colon myenteric plexus neurons, while the myenteric neurons in the sham CES group showed severely decreased synaptic vesicles.

Transmission electron microscopy was applied to observe the ultrastructure of colon myenteric plexus neurons. As shown in Figure 5, after 5 weeks of treatment, axon terminals of myenteric plexus remained in pretty good shape and contained a great deal of large dense core vesicles and small clear vesicles in the colon tissues of both control and CES treatment groups. In contrast, the two kinds of vesicles in the axon terminals of myenteric plexus decreased in the colon tissues of the sham CES group, and the nerve fibers were irregular.

These results indicated that CES might result in regeneration of the myenteric plexus neurons damaged by diphenoxylate and alosetron hydrochloride.

### Effects of CES on SYP protein expression

**Figure 6:**
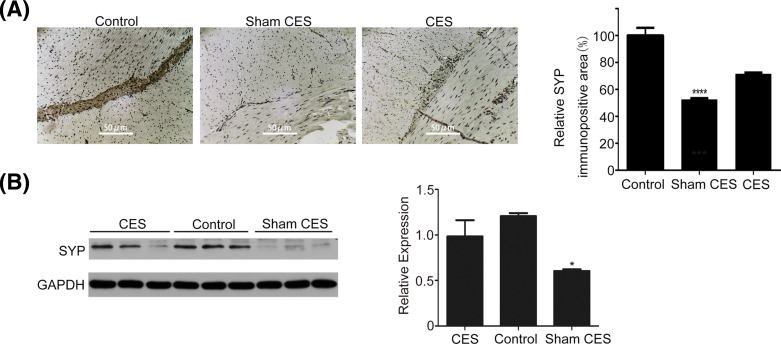
Effects of CES on SYP protein expression (**A**) Representative immunohistochemical staining of SYP. The immunopositive areas displayed brown in the regions of the myenteric plexus, and the nuclei were counterstained blue by Hematoxylin and evenly distributed across the section. The colonic intramuscular layer in both control and CES treatment groups displayed abundant SYP-immunopositive cells, while the number of SYP-immunopositive cells was decreased significantly in the sham CES group. Immunopositive areas were detected by an inverted microscope (Nikon, Japan), and its quantitative evaluation was performed by ImageJ software. The relative SYP-immunopositive area (%) (compared with the control group) was used as the y-axis. The data were represented as mean ± S.D. (****P<0.0001 versus CES). (**B**) Western blot analysis of the protein expression of SYP. The protein expression of SYP was decreased significantly in the sham CES group as compared with that of the control group, and the CES treatment restored the SYP protein expression. Densitometric values were quantitated by IQuantTL software (GE Healthcare, U.S.A.). The data were represented as mean ± S.D. (*P<0.05 versus CES).

SYP is an important calcium-binding protein with molecular weight of 38 kDa, which lies on the synaptic vesicle and mainly distributes inside the presynaptic terminals. SYP is a biomarker that reflects the neuroendocrine differentiation, therefore the down-regulated SYP expression is regarded as a symbol of nerve degeneration [26]. Immunohistochemical staining of SYP displayed that there were abundant SYP-immunopositive cells in the colonic intramuscular layer in both control and CES treatment group. However, in the sham CES group, the number of SYP-immunopositive cells was decreased significantly (Figure 6A). Furthermore, Western blot analysis showed that the protein expression of SYP was decreased significantly in the sham CES group as compared with that of the control group, and the CES treatment restored the SYP protein expression to a certain degree (Figure 6B). All of these results indicated that CES could reverse the enteric nerve degeneration caused by STC.

### Effects of CES on PGP9.5

**Figure 7:**
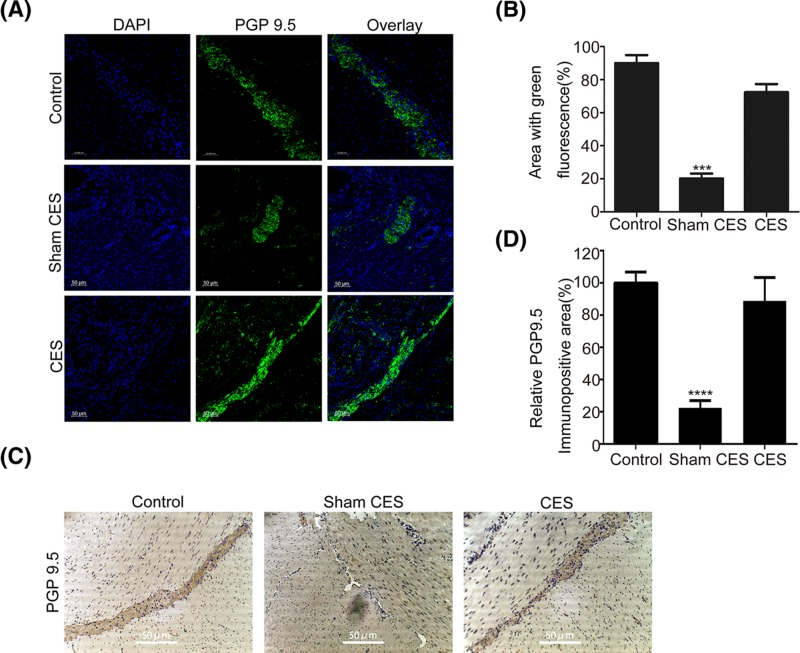
Effects of CES on PGP9.5 (**A**) The immunofluorescence of PGP9.5 observed by confocal microscopy. The immunopositive areas displayed green fluorescence, and the nuclei showed DAPI’s blue fluorescence. The sham CES group displayed little green fluorescence of PGP9.5, while both control and CES group presented remarkable green fluorescence. (**B**) The quantitative analysis was performed by ImageJ software, and the results were calculated as follows: area with green fluorescence (%) = (the area stained green by antibody/the area stained blue by DAPI) × 100(%). The data were represented as mean ± S.D. (***P<0.001 versus CES). (**C**) Representative immunohistochemical staining of PGP9.5. The immunopositive areas displayed brown in the regions of the myenteric plexus, and the nuclei were counterstained blue by Hematoxylin and evenly distributed across the section. There were a large number of PGP9.5 expressing cells in the colonic intramuscular layer in both control and CES groups, and significantly down-regulated PGP9.5-immunopositive cells in the sham CES group. Immunopositive areas were detected by an inverted microscope (Nikon, Japan). (**D**) ImageJ software was employed for the quantitative evaluation of the immunopositive areas. The relative PGP9.5-immunopositive area (%) (compared with the control group) was used as the y-axis. The data were represented as mean ± S.D. (****P<0.0001 versus CES).

PGP9.5 (ubiquitin carboxyl-terminal esterase L1, or UCHL1) is a ubiquitin hydrolase which is extensively expressed in the neuronal tissues during neuronal differentiation, thus usually used for the characterization of neurons in ENS [27]. The PGP9.5 distribution was first examined through immunofluorescence. The results in Figure 7A,B showed that both control and CES groups presented remarkable green fluorescence, while the sham CES group displayed little. Moreover, as shown in Figure 7C,D, large areas of PGP9.5 expression were present in the colonic intramuscular layer in both control and CES groups. By contrast, significantly down-regulated PGP9.5-immunopositive cells’ area appeared in the sham CES group.

### Effects of CES on CAD and S-100B

**Figure 8:**
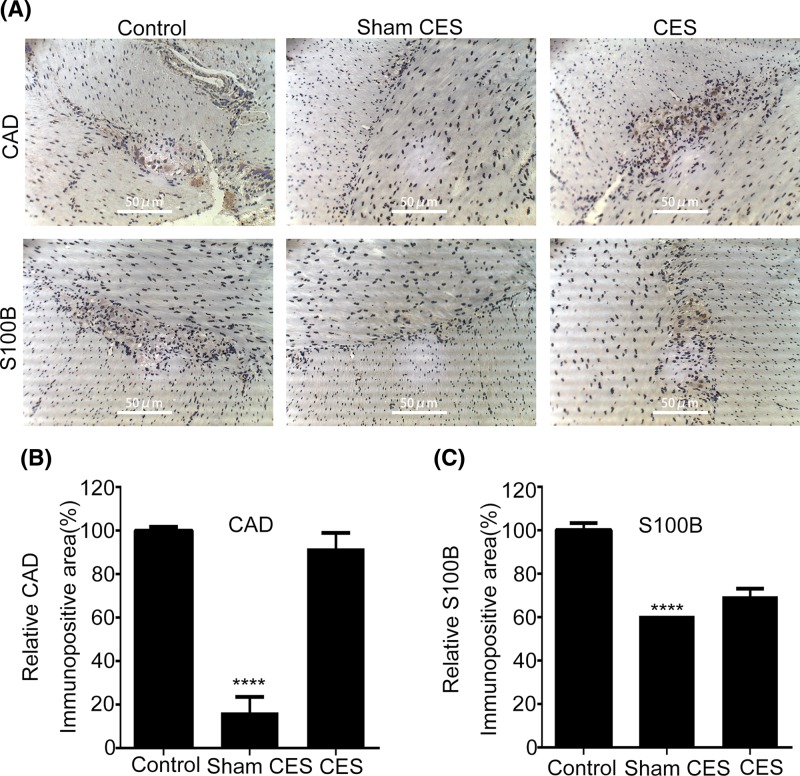
Effects of CES on CAD and S-100B (**A**) Representative immunohistochemical staining of CAD and S-100B. The immunopositive areas displayed brown in the regions of the myenteric plexus, and the nuclei were counterstained blue by Hematoxylin and evenly distributed across the section. CAD immunopositive cells in the colonic intramuscular layer of the sham CES group decreased significantly as compared with that of the control; CES recovered the expression levels of CAD in the myenteric plexus. High level of S-100B immunopositive cells appeared in the colonic intramuscular layer of the control, while the sham CES group showed significantly decreased S-100B immunopositive cells; CES recovered the expression levels of S-100B in the myenteric plexus. (**B,C**) Immunopositive areas were detected by an inverted microscope (Nikon, Japan). ImageJ software was employed for the quantitative evaluation of the immunopositive areas. The relative CAD/S100B-immunopositive area (%) (compared with the control group) was used as the y-axis. The data were represented as mean ± S.D. (****P<0.0001 versus CES).

CAD, a lysosomal protease, is chiefly expressed in ganglion cells. Immunohistochemically, different intensity of CAD staining can make a distinction between normal and abnormal neuron cells [28], and CAD has high specificity because it is hardly expressed in endothelial cells, lymphocytes and sustentacular cells [29]. S-100B is a calcium-binding protein mainly found in glial cells as well as Schwann cells which is involved in the formation of nerve fibers in the peripheral nervous system, thus can be used to assess the distribution of nerve fibers in the myenteric plexus [30]. The immunohistochemistry analysis showed that both CAD and S-100B immunopositive cells in the colonic intramuscular layer of the sham CES group decreased significantly as compared with that of the control; CES recovered the expression levels of CAD and S-100B in the myenteric plexus (Figure 8).

## Discussion

STC is a particular type of chronic constipation characterized by seriously delayed colonic transit time, which affects the old and women more frequently [31,32]. In recent years, increased interest focuses on the application of electrical stimulation in regulating gastrointestinal motility. The external electrical stimulation can alter the electrical signals generated by the natural pacemakers of gastrointestinal tract, thus improving the gastrointestinal motility and accelerate content evacuation [33]. In the past decades, direct electrical stimulation performed positive effects in animal models or humans [19,20]. Bellahsene et al. [34] reported that combined with a continuous pharmacological treatment, gastric electrical stimulation could improve gastric emptying in canine model of impaired gastric function. McCallum et al. [35] found that gastric electrical stimulation in combination with pharmacological treatment could also enhance emptying in patients with gastroparesis. Especially, gastric electrical stimulation has been approved as a clinical therapy method for gastroparesis and obesity in European and American countries [36].

The first study regarding the CES to modulate colonic motility was performed by Hughes et al. [37]. Since then, many researchers employed short-pulse CES in canine descending colon or pig cecum [20,21,38]. Researchers also applied long-pulse CES to stimulate the colon of human or animals [39]. Recently, studies showed that the prokinetic effect of pulse train CES is better than that of short-pulse CES or long-pulse CES [25]. In the present study, we first established the STC beagle model through oral administration of diphenoxylate and alosetron hydrochloride. The abdominal X-rays, colonic EMG signal, reduced defecating frequency combined with the resulting depressed emotion proved that the STC model was successfully set up. We then employed pulse train CES to treat the STC beagles through implantation of intramural electrodes in proximal colon (ileocecal site). Our study indicated that CES could enhance the colonic motility, and then accelerate the colonic content emptying. Thereafter, we investigated the underlying mechanism and presumed that CES might improve the STC symptom through the repairment of the ENS.

The neuropathy in ENS is considered to be responsible for various kinds of disordered motility including STC and the related pathophysiologic symptoms [40]. In agreement with this view, our study discovered the decreased number of ganglia in the myenteric plexus, as well as the destruction of the enteric nerve axon terminals and synaptic vesicles in the sham CES group beagles. HE staining illustrated the recovery of the ganglia between the circular and longitudinal muscles in the CES treatment group. Transmission electron microscopy also found that CES normalized the enteric neuronal vesicles including small clear synaptic vesicles and large dense core vesicles. Because the enteric neuronal vesicles contain multifarious neurotransmitters that play an important role in colonic motility function, these alterations induced by CES might prove our hypothesis that CES could enhance the colonic motility through the repairment of the ENS.

There are many proteins and enzymes such as SYP, PGP9.5, CAD and S-100B existing in the ENS that can be used as biomarkers to characterize the formation and conditions of the myenteric plexus neuron. We further explored the underlying mechanism through identification of these proteins using immunohistochemistry, Western blot analysis and immunofluorescence. SYP is a synaptic vesicle-associated protein contributing to the fusion of synaptic vesicles and the subsequent release of neurotransmitter. PGP9.5 is a neuron-specific ubiquitin hydrolase which plays an important role in protein degradation involved in neuronal differentiation, and usually acts as a specific protein for the detection of myenteric neurons. Clinical research has proved that the density of PGP9.5-positive neuronal structures in ENS was significantly decreased in colon specimens of STC patients [41]. Li et al. [26] reported that electrical stimulation could increase the SYP- and PGP9.5-positive area in the myenteric plexus in rats, and the up-regulation of SYP and myenteric neurons suggested early regeneration of the nerves and the enhanced neurotransmitter secretion which might improve the delayed gastrointestinal motility. In agreement with these studies, we also found that CES could increase the SYP- and PGP9.5-positive area in the colonic intramuscular layer in STC beagles.

Moreover, a significant up-regulation of CAD- and S-100B-positive cells was also observed in the colonic intramuscular layer in STC beagles after the CES treatment. The lysosomal protease CAD, which catalyzes the degradation of various neuropeptides, is usually found in ganglion cells of colonic myenteric plexus. S-100B is a kind of calcium-binding protein with multiple functions mainly existing in neural cells. It has been reported that both CAD and S-100B are suitable markers for the differential diagnosis of gastrointestinal motility dysfunction [28]. Thus, the overall up-regulation of SYP, PGP9.5, CAD and S-100B in the myenteric plexus might contribute to the therapeutic effect of CES on STC.

## Conclusions

The present study proves that CES with pulse trains has curative effects on the colonic motility and content emptying in STC beagles. The up-regulation of intestinal nerve related proteins such as SYP, PGP9.5, CAD and S-100B in the colonic myenteric plexus suggests that CES might reduce the degeneration of the myenteric plexus neurons, thereby producing the therapeutic effect on STC beagles. Further investigation for the underlying mechanism of nerve regeneration is necessary to better understand how CES promotes the recovery of delayed colonic motility induced by STC.

## Abbreviations

BSA: bovine serum albumin
CAD: cathepsin D
CES: colonic electrical stimulation
EMG: electromyogram
ENS: enteric nervous system
HE: hematoxylin-eosin
HRP: horseradish peroxidase
PGP9.5: protein gene product 9.5
RIPA: radio immunoprecipitation assay
STC: slow transit constipation
SYP: synaptophysin
